# Cooling Effect of Phase Change Materials Applied in Undergarments of Mine Rescuers in Simulated Utility Conditions on Thermal Manikin

**DOI:** 10.3390/ma15061999

**Published:** 2022-03-08

**Authors:** Magdalena Młynarczyk, Grażyna Bartkowiak, Anna Dąbrowska

**Affiliations:** 1 Department of Ergonomics, Central Institute for Labour Protection-National Research Institute, Czerniakowska St. 16, 00-701 Warsaw, Poland; 2 Department of Personal Protective Equipment, Central Institute for Labour Protection-National Research Institute, Wierzbowa St. 48, 90-133 Łódź, Poland; grbar@ciop.lodz.pl (G.B.); andab@ciop.lodz.pl (A.D.)

**Keywords:** protective clothing, phase change materials, PCM, mine rescuer, thermal manikin

## Abstract

The cooling effect of new undergarments (T-shirt) with PCM was measured using heat flux on a thermal manikin according to four tests variants: in T-shirt without PCM, in T-shirt with PCM, in T-shirt without PCM and with outerwear, in T-shirt with PCM and with outerwear. The tests were done in the climatic chamber under controlled conditions: t_a_ = 32 °C, RH = 70% and V_a_ = 1 m/s. The cooling effect was confirmed by thermograms taken by thermal imaging cameras located on the front and back of the manikin. The results showed that in the case of using a T-shirt with PCM, the effect of heat absorption was observed during the first several dozen minutes of operation. The mean value of the heat flux density (ΔHc) received from the manikin was +15 W/m^2^. In the case of using outerwear with a T-shirt with PCM, the mean value of the heat flux density (ΔHc) received from the manikin was +31.5 W/m^2^.

## 1. Introduction

A characteristic feature of rescue operations in coal mines is the presence of concurrent risks of explosion and climatic hazards as well as very large energy expenditure caused by the additional burden carried by the rescuers. Most frequently, the rescue work is performed in an atmosphere characterized by the following parameters: dry temperature T_s_ = (30–35) °C, relative humidity (70–85)%, air flow velocity v = (0–5) m/s. However, there are also actions carried out in extremely difficult microclimatic conditions. With large amounts of methane burning and the presence of smoky air, it was found that the dry temperature was T_s_ = (35–45) °C, relative humidity (70–97)%, and the air flow velocity v = (0.5–2.5) m/s [1,2,3].

The most common cause of fatal accidents involving mine rescuers in Poland (40% of the total number) is the risk of methane, fire gases, and coal dust explosion, and the main cause of deaths among rescuers are thermal burns to a significant body surface area and to the respiratory tract. However, the second most frequent cause of fatal accidents in rescuers during rescue operations is difficult microclimate conditions, causing overheating of rescuers’ bodies and death from heat stroke (26% of the total number of deaths). The thermal load on mine rescuers caused by effort in very difficult microclimate conditions additionally increases due to their equipment, consisting of complete clothing (6 kg), respiratory protection equipment (15 kg), lamp, and other equipment, being an additional load of 25 kg on average. When it is necessary to transport injured persons, the average additional load is 45–50 kg/person [2,4]. Dry, complete clothing constitutes 25% of the basic load of the rescuer, and this load increases during work, because getting wet (or crossing water lagoons) causes an additional load, increasing energy expenditure.

The safety of mine rescuers largely depends on the equipment used, which also includes protective clothing. When developing this, it is necessary to take into account all the risks to which the mine rescuer is exposed, i.e., explosion and fire hazards, as well as its impact on thermal comfort. Therefore, the challenge is to develop a clothing set that will protect against threats and meet all the essential requirements as well as support the body’s thermoregulation, thanks to the use of appropriately selected materials.

Such conditions and risks require the use of garments that include additional cooling elements. Their design should not eliminate the possibility of draining and evaporation of sweat, because this ensures an effective heat dissipation process in extremely high temperatures. Taking into account the fact that, during rescue operations, an increase in body and skin temperature is observed due to the significant thermal load of the rescuer, it is necessary to collect excess heat from the rescuer’s body; phase change materials may be suitable for this purpose. Phase change materials absorb, store, and release large amounts of energy in the form of latent heat, in the temperature range of phase transformation (i.e., melting and crystallization temperatures) [5].

Accordingly, within the framework of the strategic research project “Improving safety in mines” conducted in Poland, it was undertaken to develop sets of protective undergarments and clothing for mine rescuers to ensure the safety of their operations and the possibility of removing excess heat through the application of phase change materials (PCMs).

Considering the human-clothing-environment system as a whole, it can be concluded that there are many factors that can influence the effectiveness and duration of a specific heat effect. These factors are PCM heat capacity, amount of PCM, phase transition temperature range, temperature gradient between human skin and PCM melting/crystallization point, structure of the textile substrate on which the PCM materials are applied, body area covered by the PCM textile material, layer configuration material with PCM in relation to the skin, and pre-conditioning of PCM before use (i.e., complete crystallization or melting of the PCM) [6,7].

The overall heat capacity of the PCM in a given product depends on the specific heat capacity of the PCMs used and their quantity. In recent years, significant progress in PCM incorporation in textiles can be observed. Fifteen years ago, only 20% by weight of PCM could be incorporated into fibers, flat textiles, or foams, which unfortunately ensured a short cooling or heating period [6]. The current state of the art indicates that ~83 wt.% of PCM can be incorporated into the tubular polypropylene filament [8]. Teunissen et al. [9] analyzed the influence of the kind of PCM as well as its packaging material and segmentation on cooling capacity, duration, and power in terms of ensuring cooling to the human body in heat-stress conditions. The researchers came to conclusion that a lower melting point of PCM is related to a higher cooling power and shorter cooling duration. Moreover, they stated that an insulative layer in a cool pack considerably extends the cooling duration but decreases the average cooling power. However, thanks to the higher possible weight of PCM in packs than PCM incorporated into fibers, the use of packs with PCM is a good alternative in order to increase the efficiency and duration of the thermal effect [10,11].

The purpose of this publication is to present the results of work carried out under the aforementioned project, related to modeling undergarments containing PCM, with the function of microclimate thermoregulation, designed for mine rescuers.

## 2. Materials and Methods

In order to ensure the safety of mine rescuers, they should be equipped with protective clothing meeting essential health and safety requirements of Regulation (EU) 2016/425 [12] for personal protective equipment. Due to a risk of exposure to thermal factors and explosion, this clothing should meet specific requirements of relevant standards, i.e., EN ISO 11612:2015 [13] in relation to protection against heat and flame, and EN 1149-5:2018 [14] in relation to electrostatic dissipation properties [15]. Moreover, taking into account the thermal load that mine rescuers are exposed to during rescue operations, their clothing should support thermoregulation processes, be made of low-weight fabrics, and be characterized by low thermal and water vapor resistance in order to allow dry and wet heat exchange. Therefore, undergarments with phase change materials meeting the above-mentioned requirements were developed and tested in the study. 

The undergarments were designed to be used together with protective clothing and the closed-circuit breathing apparatus PSS BG-4 EP, often used by mine rescuers.

### 2.1. Tested Object

#### 2.1.1. Phase Change Materials

Phase change materials (PCM) absorb, accumulate, and release considerable amounts of energy in the form of latent heat, within the range of phase change temperatures (melting and solidifying points). In order to allow for drawing the excess heat from the human body, the melting process causing heat absorption is used [16,17]. Therefore, special attention was paid to the proper selection of PCM for integration within the protective undergarments for mine rescuers in accordance with the predicted utility conditions in terms of the following two most important requirements: (i) gradual melting process in order to provide their prolonged operation time, and (ii) high latent heat during melting in order to ensure the highest possible cooling efficiency. On the basis of the analysis of rescue operations in coal mines, the selection of two kinds of PCM differing in their melting temperature (32 and 37 °C) was made. Selected PCMs were characterized by an enthalpy of 160–190 J/g, which ensures cooling in an ambient temperature of about 30 °C to 40 °C. In order to introduce PCM macrocapsules into T-shirts, cooling elements in the form of bags with channels filled with the macrocapsules were designed.

#### 2.1.2. Protective Undergarments with PCM

**Figure 1:**
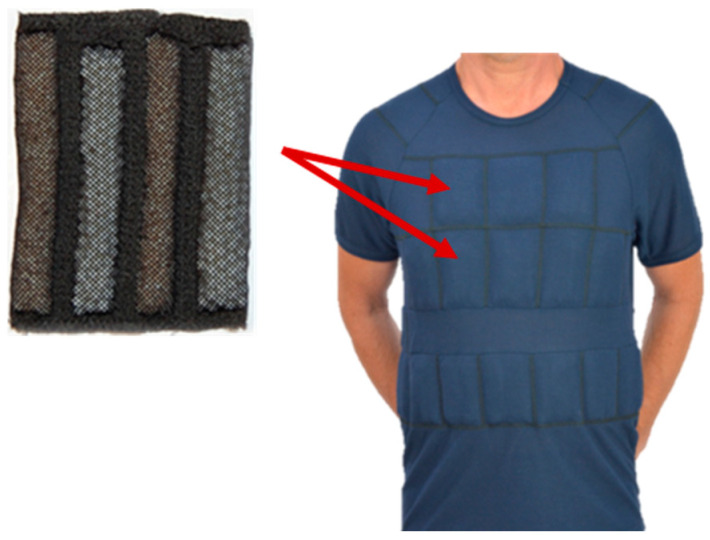
A view of protective T-shirt with PCM elements.

The protective undergarments (Figure 1) were made from a knitted fabric with the following composition: 90% Lenzing FR, 8% p-aramid, 2% antistatic fibre, and mass per square meter of 193.5 g/m^2^. The undergarments have the form of a T-shirt and shorts.

The developed protective undergarments were equipped with PCM cooling elements (bags), integrated by means of specially designed pockets. Their distribution in the undergarments was designed considering the body areas that are subjected to the greatest heating, as well as the requirement of compatibility with other rescue equipment (in particular, the breathing apparatus). Therefore, the location of PCM cooling elements in the undergarments includes only pressure-free areas in order to avoid any inconvenience due to their implementation. The total weight of the PCM integrated with the undergarments was equal to 860 g. Additionally, PCM cooling elements were subjected to a flammability test, which confirmed their conformance with the requirements of EN ISO 11612:2015 [13].

#### 2.1.3. Protective Clothing for Mine Rescuers

In order to provide whole body protection to mine rescuers, the outer layer of protective clothing for mine rescuers consists of a top shirt and waist-high bib and trousers with braces. A woven fabric made of 93% meta-aramid fibers, 5% para-aramid fibers, and 2% antistatic fibers was selected for the protective clothing. Similarly, as in the case of the undergarments, a crucial feature of the developed clothing is the compatibility of its design with the equipment used by mine rescuers. In this case, pocket arrangement considered potential contact with other equipment. In order to increase the cooling efficiency, the protective clothing was also equipped with PCM elements on the stand-up collar and on the back of the top shirt. Moreover, both on the shirt and the trousers, several vents were incorporated in order to support drawing the excess heat from the rescuer’s body. 

**Figure 2:**
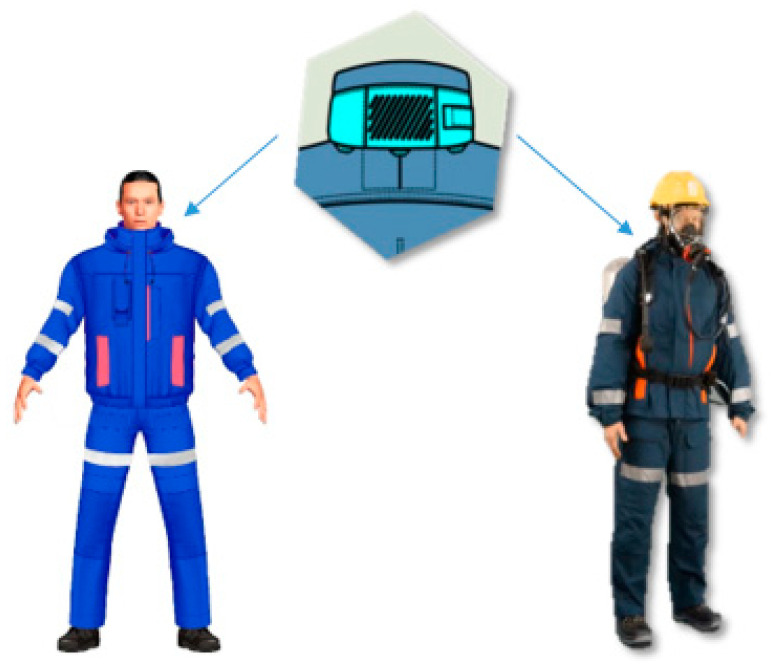
A view of protective clothing with PCM elements on the collar.

A view of the developed protective clothing for mine rescuers is presented in Figure 2.

### 2.2. Research Equipment

#### 2.2.1. Newton-Type Thermal Manikin 

A full-size male Newton-type manikin consisting of 34 segments (Measurement Technology Northwest, Seattle, WA, USA) was used. The design of the manikin allows both dry and wet heat transfer tests to be carried out. A detailed description of the manikin is contained in [18,19].

#### 2.2.2. Climatic Chamber

In the climatic chamber (Weiss, type WK23), tests can be carried out in controlled conditions in the range of air temperature from −40 °C to 70 °C, relative humidity from 10% to 90%, and air velocity up to 3 m/s [20].

#### 2.2.3. Microclimatic Meter 

The thermal Comfort Data Logger 1221 INNOVA and Indoor Climate Analyzer 1213 (Bruel & Kijær) were used for controlling the parameters in the climatic chamber.

#### 2.2.4. Thermal Imaging Cameras 

Professional portable thermal imaging cameras ThermaCAM SC660 and T660 (FLIR Systems) were used for the tests. They allow the registration of thermograms with a resolution of 640 × 480 pixels, with a frequency of 30 Hz.

### 2.3. Methodology

In order to determine the effectiveness of cooling undergarments, the density of heat fluxes in the tested sets of clothing was calculated for 4 performed test variants (Table 1).

In order to determine the temperature distribution on the surface of the tested clothing, thermal imaging cameras were used. These cameras were located on the front (FLIR T660) and on the back of the manikin (FLIR SC660) to monitor temperature changes on the tested surfaces at the same time.

The manikin’s skin temperature for the undergarment test was 35.0 °C (variants I–II) and with the outerwear was 35.5 °C (variants III–IV). The adopted values were based on previously performed studies on a group of volunteers [21]. The surface temperature of the manikin was controlled by the ThermDAC program, dedicated to the thermal manikin (type Newton). The manikin operated in the mode of maintaining a constant temperature of the manikin’s surface with variable power consumption. The temperature is controlled by a closed loop inside the shell of manikin. Imbedded temperature sensor wires report the skin temperature to the zone controller, which controls the zone heater wire accordingly, to keep the skin temperature at the set point temperature (within ±0.1 °C). 

On the basis of studies with volunteers [21], the sweat rate was set at 500 mL/h·m^2^ for undergarments and 600 mL/h·m^2^ for a set of clothes.

**Figure 3:**
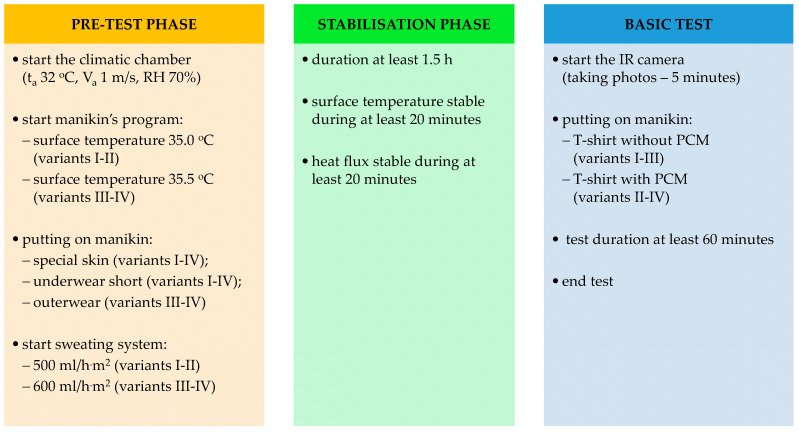
Test scheme.

The heat flux density tests were carried out in controlled, identical conditions in a climatic chamber (32 °C, 70%, 1 m/s). These conditions were selected on the basis of previous tests [21]. The study scheme is shown in Figure 3.

**Figure 4:**
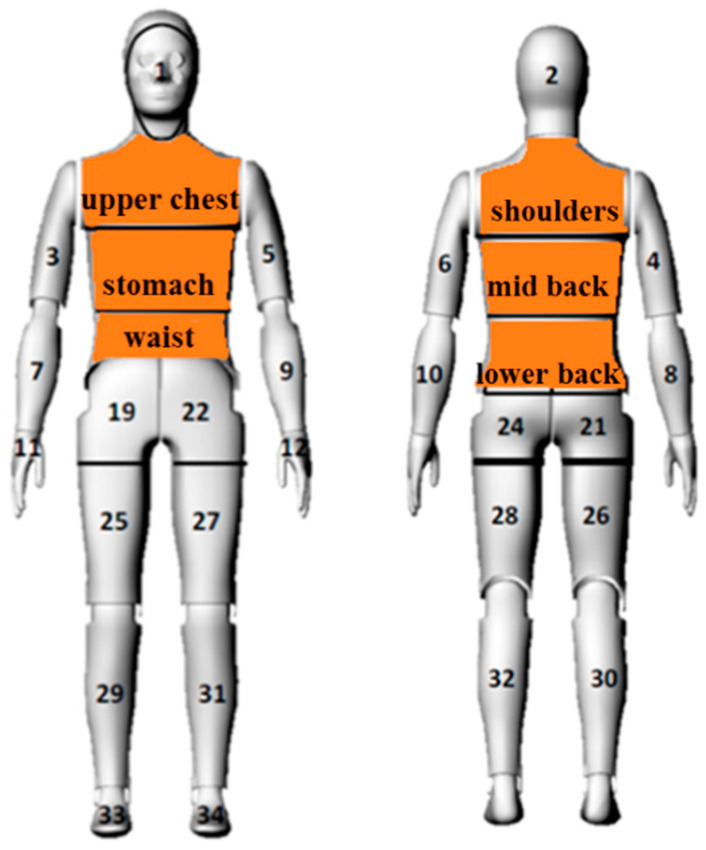
Manikin segments subjected to heat flux density analysis.

The heat flux densities obtained from 6 manikin segments were analyzed—upper chest, shoulders, stomach, mid back, waist, and lower back—on which the tested undergarments had a direct impact (Figure 4).

Before the test, the undergarments and outerwear were in the laboratory (in unfolded condition). The temperature values in the laboratory were controlled to avoid activation of the PCM. The average air temperature in the laboratory was 24 ± 2 °C.

**Figure 5:**
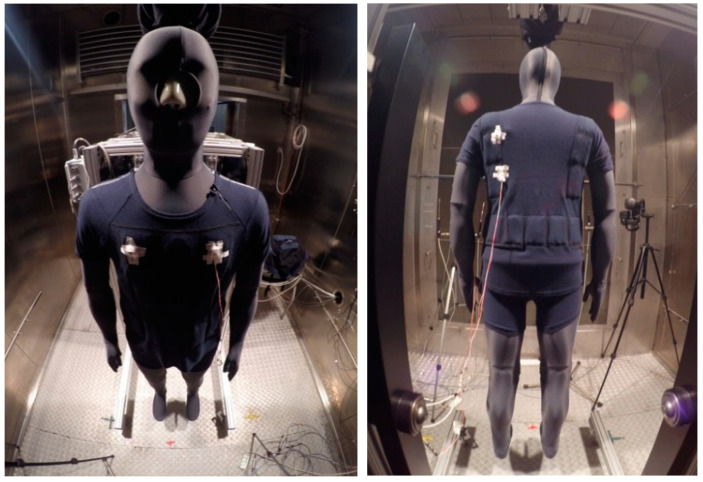
The manikin wearing type A undergarments.

The sample photos from the tests of type A undergarments in the climatic chamber (variants I and II) are shown in Figure 5.

**Figure 6:**
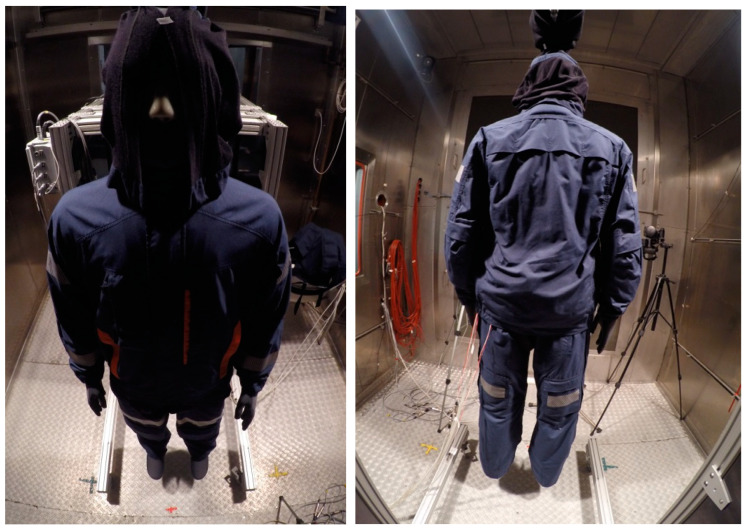
The manikin wearing type A undergarments with outerwear.

The sample photos from the tests of type A undergarments with outerwear in the climatic chamber (variants III and IV) are shown in Figure 6.

## 3. Results

The results of the research on heat flux density and illustrative photos from the thermal imaging cameras are presented below.

### 3.1. Results of Heat Flux Density

#### 3.1.1. Heat Flux Density for Undergarments A (Variants I and II)

**Figure 7:**
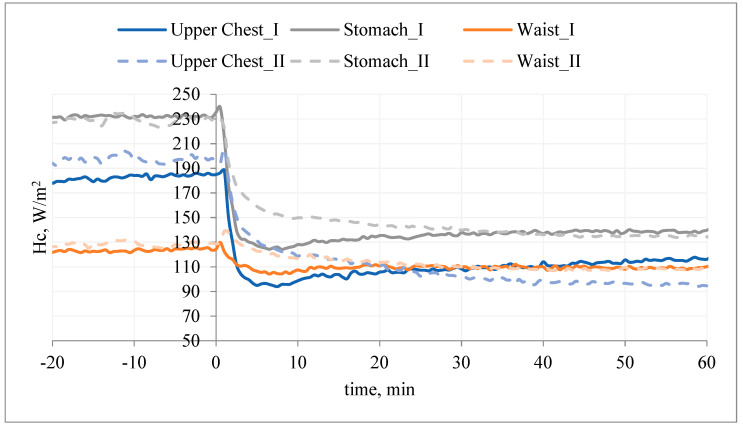
An example of the heat flux density, Hc, on selected manikin segments (chest) during testing of type A undergarments without PCM (variant I) and with PCM (variant II).

Putting on the undershirt (without PCM) resulted in a reduction of the heat flux density (ΔHc_I) in relation to the initial stabilization. Putting on the undershirt with PCM also reduced the heat flux density (Hc_II), but only in the first minutes of the study (mainly on the chest segment). The heat flux density with PCM (Hc_II) was higher than in the variant without PCM (Hc_II > Hc_I; ΔHc_II < ΔHc_I) (Figure 7).

The heat flux density on different body parts (before putting the T-shirt on the manikin) depends on, inter alia, the construction of the garment (used materials), the shape of the manikin (garment is differently fitted to manikin’s shell for different body parts, resulting in different sizes of air gaps), and the air flow patterns around the manikin. Resistance against the heat loss in different amounts at different body parts causes a difference in heat loss amount. The changes of heat loss for different body parts after putting on the T-shirt without PCM results in disturbance of the steady state. The size/level of changes depends on fitting the T-shirt to the manikin’s shell, the size of the air gaps, and the construction of the T-shirt. Some parts of T-shirt have stitching or double fabric. The changes of heat loss for different body parts after putting on the T-shirt with PCM depend on the reasons mentioned above, and the PCM influences the cooling effect. It should be noted that the PCMs were not evenly distributed over the entire surface of the T-shirt (Figure 1).

It can be concluded that the applied PCM caused a decrease in the surface temperature of the manikin and thus an increase in Hc. The use of PCM resulted in milder changes in Hc_II over time. After about 30 min of the examination (on the chest), the opposite relationship was observed: Hc_II < Hc_I (ΔHc_II > ΔHc_I). It can be assumed that after this time, the pockets with PCM were an additional insulating layer, which caused an increase in temperature on the manikin’s surface (and thus a decrease in heat flux density).

**Figure 8:**
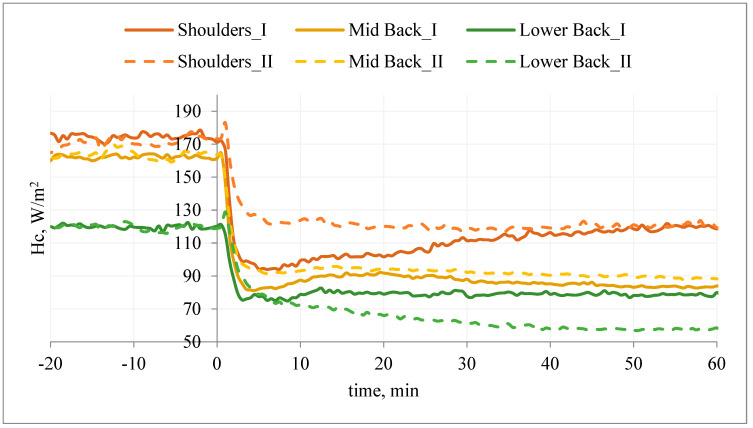
An example of the heat flux density, Hc, on selected manikin segments (back) during testing of type A undergarments without PCM (variant I) and with PCM (variant II).

A similar situation was observed on the back side of the manikin (Figure 8).

Application of the undershirt (without PCM) reduced the heat flux density (ΔHc_I) in relation to the initial stabilization. Putting on the T-shirt with PCM also resulted in a reduction of the heat flux density (Hc_II), but in the first minutes of the test (5–15 min), the heat flux density (Hc_II) was higher than in the variant without PCM (Hc_II > Hc_I, ΔHc_II < ΔHc_I) (Figure 7 and Figure 8). It can be concluded that the applied PCM decreased the temperature of the manikin surface and thus increased Hc. The use of PCM resulted in the changes of Hc_II over time being milder (segment: shoulders, mid back). After about 5–15 min of the test (segment: lower back and shoulders), the opposite relationship was observed: Hc_II < Hc_I (ΔHc_II > ΔHc_I). It could be assumed that after this time the pockets with PCM were an additional insulating layer, which caused an increase in temperature on the surface of the manikin (and thus a decrease in heat flux density).

**Figure 9:**
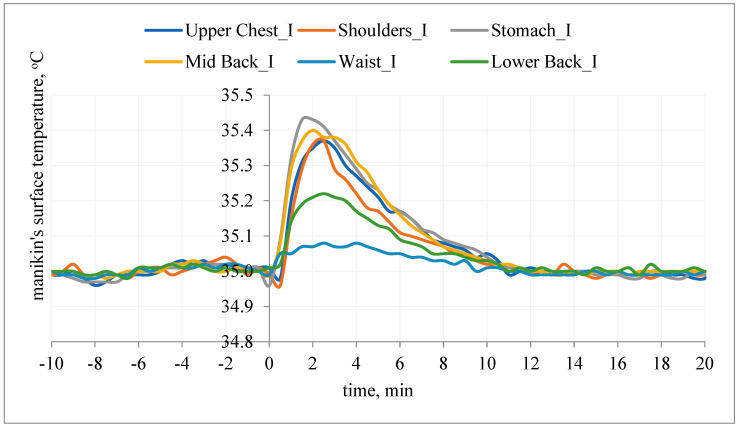
The manikin’s surface temperature after application of the undershirt without PCM (variant I).

**Figure 10:**
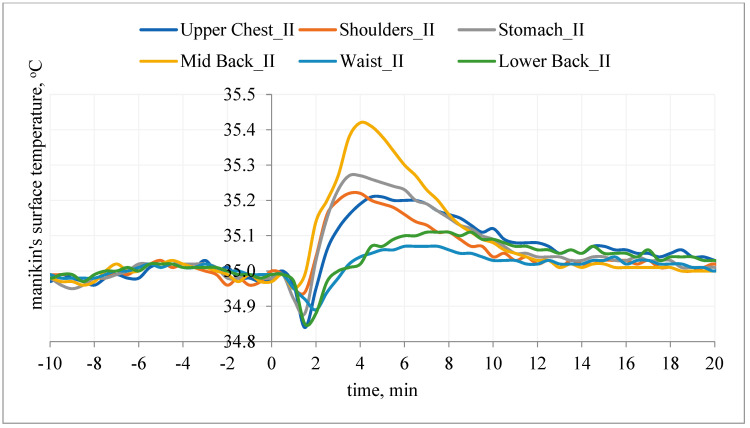
The manikin’s surface temperature after application of the undershirt with PCM (variant II).

After putting on the undershirt, the surface temperature response of the manikin on the chest and on the back were also observed (Figure 9 and Figure 10).

The surface temperature (before putting the T-shirt on manikin) was constant (research assumptions) at 35 °C, the steady-state condition. After application of the undershirt without PCM, an increase in surface temperature of 0.1 °C (waist segment), 0.2 °C (lower back segment) and 0.4 °C (upper chest, shoulders, stomach, mid-back segments) was observed (Figure 9). The changes in surface temperature after application of the T-shirt without PCM follow from a disturbance of the steady state. The variation in values may be due to the T-shirt fitting the manikin’s shape.

After application of the T-shirt with PCM, the manikin’s surface temperature dropped from the assumed value of 35 °C by about 0.2 °C, and then saw an increase from the assumed value of 35 °C of 0.1 °C (waist, lower back segments), 0.25 °C (upper chest, shoulders, stomach), and 0.4 °C (mid-back segment). The difference in surface temperature after application of the T-shirt with PCM followed from a disturbance of the steady state and depended on the construction of T-shirt, the cooling effect of PCM, and the localization of PCMs.

After analyzing the heat flux density curves during the test, the period of 20 min from application of the T-shirt on the manikin was taken into account for further analyses.

The cooling effect of PCM (ΔHci_cool (0–20)) within 20 min of putting on the T-shirt was calculated as

ΔHci_cool(0–20) = ΔHci_T-shirt(0–20)_variant II − ΔHci_T-shirt(0–20)_variant I

ΔHci_T-shirt(0–20) = Hci_(0–20)_variant I − Hci_(−20–0)_variant I

where

- ΔHci_T-shirt(0–20): change in the average density of heat flux caused by the T-shirt, for 20 min from the moment of application of the T-shirt, compared to the average Hc value from 20 min application of the T-shirt (from pre-test at stabilization state) (variant I), W/m^2^;
- ΔHci_cool(0–20): difference in heat flux density changes between the variant with active cooling (variant II) and the variant without active cooling (variant I), W/m^2^.

**Figure 11:**
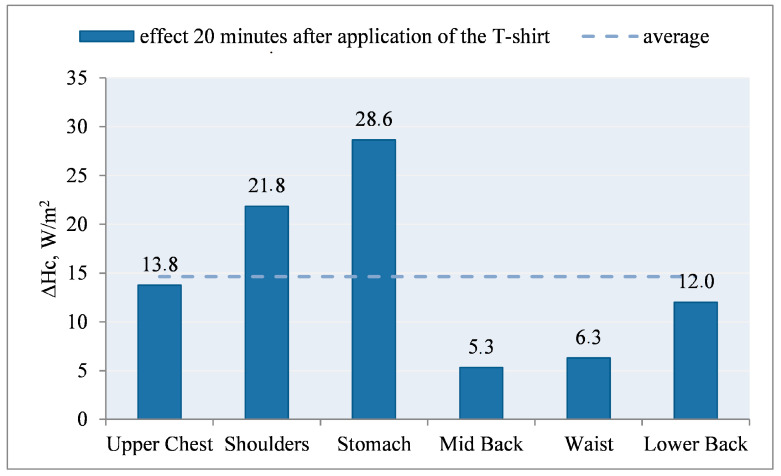
Influence of PCM elements on the thermal manikin: difference in the density of heat fluxes ΔHci_cool 20 min after application of T-shirt on the selected manikin segments for the values obtained for variants I and II.

The calculated influence of the PCM elements on the ΔHci_cool thermal manikin is shown in Figure 11.

After use of PCM elements in the first 20 min of the study, an increase in the density of heat flux (ΔHc) was noted, with the largest seen in the places where the PCM elements were the most numerous and they adhered to the shell of the manikin: stomach, shoulders, upper chest (i.e., mainly chest and upper body of the manikin). By averaging the obtained results, it can be seen that the PCM elements had an average of ~|15|W/m^2^.

#### 3.1.2. Heat Flux Density for Undergarments (Type A) with Outerwear (Variants III and IV)

**Figure 12:**
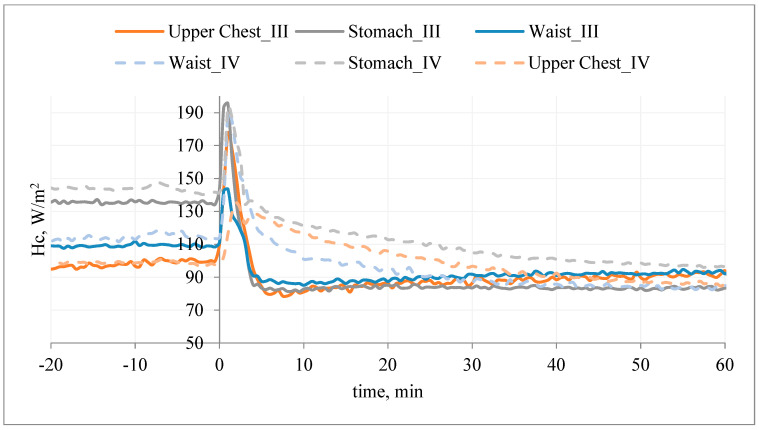
An example of the heat flux density, Hc, on selected manikin segments (chest) during the test of T-shirt A without PCM together with outer garments (variant III) and T-shirt A with PCM together with outer garments (variant IV).

An exemplary course of the heat flux density during the study of the T-shirt, type A, without PCM and with outerwear (variant III) and the T-shirt, type A, with PCM and with outerwear (variant IV) are shown in Figure 12.

Application of the T-shirt A without PCM under the outer clothing resulted in a reduction of the heat flux density (Hc_III) in relation to the initial stabilization. Application of the T-shirt A with PCM under the outer clothing also resulted in a reduction of the heat flux density (Hc_IV), but in the first minutes of the study (mainly on the chest), the heat flux density (Hc_IV) was higher than in the variant without PCM (Hc_IV > Hc_III ΔHc_IV < ΔHc_III) (Figure 12). It can be concluded that the applied PCM decreased the temperature of the manikin surface and thus increased Hc.

The use of PCM resulted in milder changes in Hc_IV over time. After about 40 min of the examination (upper chest and waist segments), the opposite relationship was observed: Hc_IV < Hc_III (ΔHc_IV > ΔHc_III). It can be assumed that after this time the PCM T-shirt on these segments (excluding the stomach segment) is an additional insulating layer, causing an increase in temperature on the surface of the manikin (and thus a decrease in heat flux density) (Figure 12).

**Figure 13:**
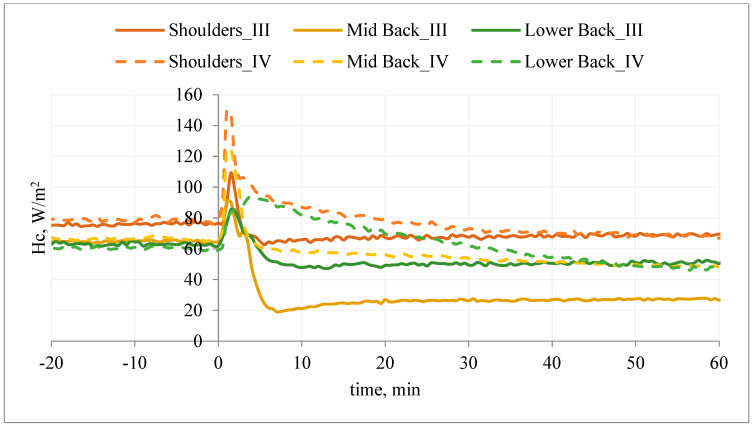
An example of the heat flux density, Hc, on selected manikin segments (back) during the test of T-shirt A without PCM together with outer garments (variant III) and T-shirt A with PCM together with outer garments (variant IV).

A similar situation was observed on the back of the manikin (Figure 13). The application of the T-shirt resulted in a reduction of the heat flux density (ΔHc_III) in relation to the initial stabilization. Wearing T-shirt A with PCM under the outer clothing also resulted in a reduction of the heat flux density (Hc_IV); however, in the first 40 min of the study, the heat loss density (Hc_IV) was higher than in the variant without PCM (Hc_IV > Hc_III; ΔHc_IV < ΔHc_III) (Figure 13).

It can be concluded that the applied PCM caused a decrease in the temperature of the manikin surface and thus an increase in Hc. The use of PCM resulted in milder changes in Hc_IV over time. After about 40–45 min of the study (shoulder and lower back segments), the inverse relationship was observed: Hc_IV < Hc_III (ΔHc_IV > ΔHc_III). It can be assumed that after this time the PCM jacket on these segments (excluding the mid-back segment) was an additional insulating layer, causing an increase in temperature on the surface of the manikin (and thus a decrease in the density of heat loss streams).

**Figure 14:**
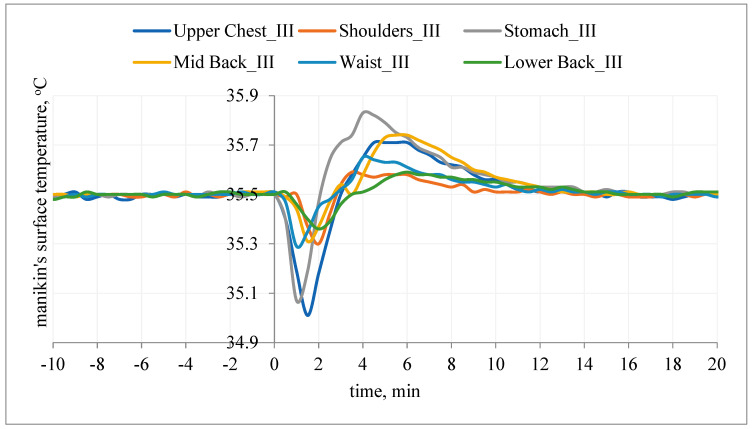
The surface temperature of the manikin after application of T-shirt A without PCM under the outer clothing (variant III).

**Figure 15:**
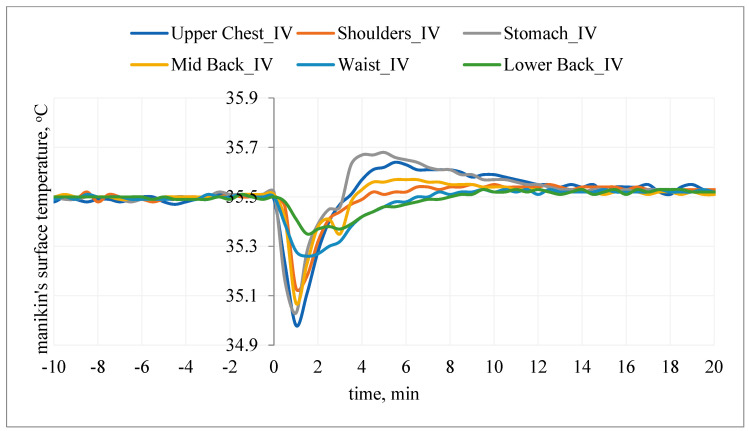
The temperature of the manikin’s surface after application of T-shirt with PCM under the outer clothing (variant IV).

After the T-shirt was put on, the temperature responses of the manikin’s surface on the chest and on the back were also observed (Figure 14 and Figure 15).

After application of T-shirt A without PCM under the outer clothing, at the beginning, the temperature of the manikin’s surface decreased from the assumed value of 35.5 °C by approx. 0.5 °C (chest, stomach segments) and approx. 0.3 °C (other segments), and then increased from the assumed value of 35.5 °C by approx. 0.1 °C (waist segment, lower back, shoulders) and 0.25–0.33 °C (stomach, upper chest, mid-back segments). The differences in values may result from the T-shirt fitting the manikin’s shell (Figure 14).

After application of T-shirt A with PCM under the outer clothing, a decrease in the temperature of the manikin’s surface from the assumed value of 35.5 °C by approx. 0.5 °C (upper chest, shoulders, stomach, mid-back segments) and approx. 0.2 °C (waist, lower back segments) was observed at the beginning, followed by an increase from the assumed value of 35.5 °C by max. 0.17 °C (stomach segment). The use of PCM resulted in a smoother course of temperature changes on the surface of the manikin (Figure 15).

Based on the above dependence, it can be said that in the period of 20 min after application of the T-shirt with PCM under the outer clothing, the value of heat flux density increased in relation to the T-shirt without PCM. 

**Figure 16:**
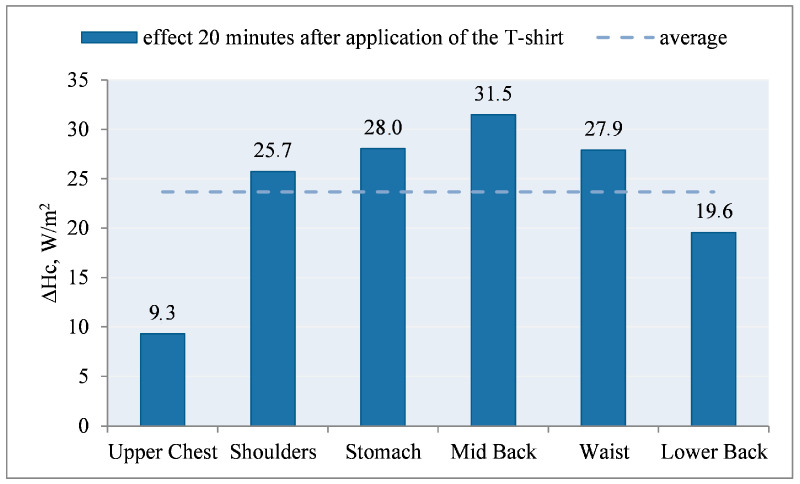
Influence of PCM elements on the thermal manikin: difference in the density of heat fluxes ΔHci_cool 20 min after application of T-shirt A under the outer clothing on the selected manikin segments for the values obtained for variant IV and III.

Thus, we observed the desired heat reception from the thermal manikin through the cooling elements (Figure 16).

The cooling effect of PCM (ΔHci_cool (0–20)) within 20 min of putting on the T-shirt was calculated as

ΔHci_cool(0–20) = ΔHci_T-shirt(0–20)_variant IV − ΔHci_T-shirt(0–20)_variant III

ΔHci_T-shirt(0–20) = Hci_(0–20)_variant III − Hci_(−20–0)_variant III

where:

- ΔHci_T-shirt(0–20): change in the average density of heat flux caused by the T-shirt, 20 min from the moment of application of the T-shirt, compared to the average Hc value from 20 min before application of the T-shirt (from pre-test at stabilization state) (variant III), W/m^2^;
- ΔHci_cool(0–20): difference in heat flux density changes between the variant with active cooling (variant IV) and the variant without active cooling (variant III), W/m^2^.

The calculated effect of the PCM elements on the ΔHci_cool thermal manikin is shown in Figure 16.

After the use of PCM elements, under the outerwear, in the first 20 min of the study, an increase in the density of heat fluxes (ΔHc) was noted, with the highest in the following segments: mid back, stomach, waist, shoulders, lower back. By averaging the obtained results, it can be stated that the PCM elements had an average of ~|31.5|W/m^2^.

### 3.2. Illustrative Photos Taken by Thermal Imaging Cameras

#### 3.2.1. Thermograms of the Undershirt (Variants I and II)

Below are the illustrative photos taken with the FLIR T660 IR camera (photos of the manikin’s chest) and the FLIR SC660 camera (photos of the manikin’s back). The following assumptions were made for all photos: ε = 0.98 (like human skin), t_a_ = 32 °C, RH 70%.

The photos were for reference only, in order to observe the changes that occurred during the application of the newly developed undergarments. Pictures from thermal imaging cameras are presented below, according to the following scheme: a photo before application of the tested T-shirt, after application of the T-shirt, and after 10 min of testing (Table 2).

The above photos show the influence of the PCMs used. The places of application of PCM are shown. After approx. 10 min of the test, a drop in temperature was observed on the surface of the t-shirt in the place where the PCM had been placed, by approx. 2 °C. 

#### 3.2.2. Thermograms of the Outerwear (Variants III and IV)

Below are the illustrative photos taken with the FLIR T660 IR camera (photos of the manikin’s chest) and the FLIR SC660 camera (photos of the manikin’s back). The following assumptions were made for all photos: ε = 0.98 (like human skin), t_a_ = 32 °C, RH 70%.

The photos taken were for reference only, in order to observe the changes that occurred during the application of the newly developed undergarments with outerwear.

Photos from thermal imaging cameras are presented below, according to the following scheme: a photo before application of the T-shirt, after application of the T-shirt, and after 10 min of testing (Table 3).

After the use of the T-shirt with PCM, a decrease in temperature on the surface of the outerwear by approx. 2.5 °C on the chest was observed. The use of the T-shirt with PCM under outer clothing caused a decrease in temperature on the surface of the outerwear (at shoulder height, in the place where the PCM was located) by approx. 4 °C.

## 4. Discussion and Conclusions

In order to investigate the density of heat fluxes during the use of the newly developed undergarment type A, the research methodology was developed. The temperature of the manikin’s surface and the intensity of sweating were planned on the basis of the results of studies with the participation of volunteers [21]. Four test variants were used: undergarment A without PCM (variant I), undergarment A with PCM (variant II), undergarment A without PCM with outerwear; (variant III), undergarment A with PCM with outerwear (variant IV). Simulations with the use of a manikin were carried out at an ambient temperature of 32 °C, relative humidity ~70%, and an air velocity of 1.0 m/s.

After analyzing the value of the heat flux density, the manikin’s surface temperature, the temperature of the fabric coating, and the temperature of the linen surface, it can be concluded that the use of the T-shirt A with PCM had a positive effect on the heat reception from the manikin. A deeper analysis of the heat flux density showed the desired effect in the first 20–30 min of the test after wearing the T-shirt with PCM. The mean value of the heat flux density (ΔHc) received from the manikin (observed as an increase in Hc) was +15 W/m^2^. Application of a PCM t-shirt, in the first 20 min, caused a decrease in the temperature of the manikin’s surface, mainly on the chest.

In the case of using outerwear with the undergarment A with PCM, a positive effect of cooling elements on heat loss from the surface of the manikin was observed. The analysis of the course of the heat flux density showed the desired effect in the first 20–30 min of the test after application of the PCM T-shirt. The mean value of the heat flux density (ΔHc) received from the manikin (observed as an increase in Hc) was +31.5 W/m^2^. Application of a PCM T-shirt, in the first 20 min, caused a decrease in the temperature of the manikin’s surface, mainly on the chest.

The above results show that, in the case of using a T-shirt with PCM, the effect of heat absorption is observed during the first several dozen minutes of operation. After about 40 min, the PCM inserts constituted an additional insulating layer. It should be noted, however, that these are the results for tests carried out in one variant of the ambient temperature: 32 °C, for the temperature of the manikin shell in the range of 35.0 °C to 35.5 °C. In undergarment A, an equilibrium mixture of MPCM 32 and MPCM 37 macrocapsules was used. In the case of MPCM 32, the melting process takes place in the temperature range of approx. (25–32) °C (maximum of approx. 30 °C, ΔHt 83.6 J/g), while in the case of MPCM 37 macrocapsules, the melting process takes place in the temperature range of approx. (33–44) °C (maximum of approx. 39 °C, ΔHt 175.1 J/g). Therefore, there is a supposition that in such research conditions, the phase changes in MPCM 37 macrocapsules might not have occurred. Moreover, it is worth pointing out that due to the extremely burdensome conditions of rescue actions in coal mines (high environmental temperature, high relative humidity, high load of additional equipment, high physical expenditure), the time of operations is limited and corresponds to the above-mentioned time of cooling. Alternatively, when a longer time of cooling is needed and the conditions are not so burdensome, other solutions for protective undergarments with cooling functions may be used, based on the air ventilation method [22].

The time of the cooling effect delivered by PCMs is one of the shortcomings of this cooling method. Therefore, research works aimed at development of fabrics with PCMs characterized by high enthalpy, as well as a wide scope of melting temperatures in the range close to the temperatures in the undergarment microclimate, should be continued in order to provide a prolonged time of cooling. Moreover, specific needs related to potential application, such as flame retardancy, resistance to heat, and electrostatic dissipation properties, should be also considered. Integration techniques should be also further explored in order to guarantee a high level of ergonomic properties of such kinds of clothing.

Illustrative photos from thermal imaging cameras confirmed the above dependencies and showed the operation of cooling elements.

## Figures and Tables

**Table 1:** Test variants.

Variant	Undergarments	Outerwear	Cooling System
I	Undergarments Type A	− without	OFF (without PCM)
II		− without	ON (with PCM)
III		+ with	OFF (without PCM)
IV		+ with	ON (with PCM)

**Table 2:** Pictures from thermal imaging cameras for variants I and II.

**Variant I_T-Shirt without PCM**		
Before putting on T-shirt	Directly after putting on T-shirt	10 min after the putting on T-shirt
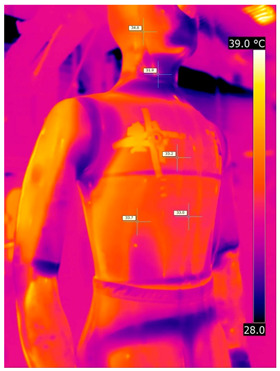	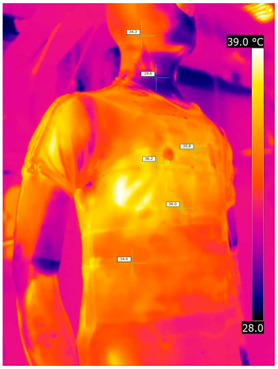	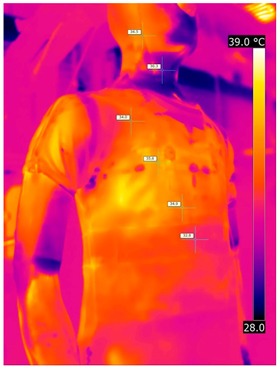
**Variant II_ T-Shirt A with PCM**		
Before putting on T-shirt	Directly after putting on T-shirt	10 min after the putting on T-shirt
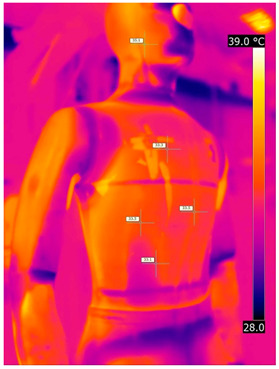	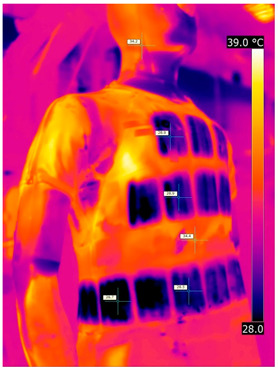	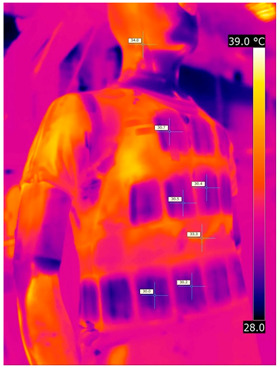
**Variant I_T-Shirt without PCM**		
Before putting on T-shirt	Directly after putting on T-shirt	10 min after the putting on T-shirt
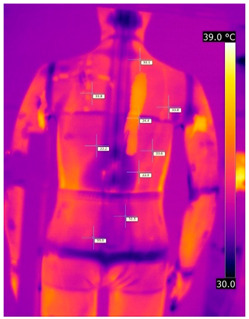	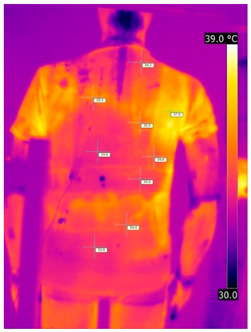	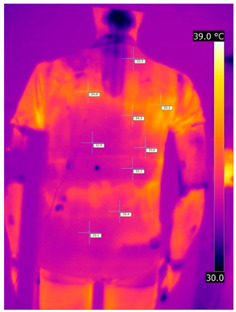
**Variant II_T-Shirt A with PCM**		
Before putting on T-shirt	Directly after putting on T-shirt	10 min after the putting on T-shirt
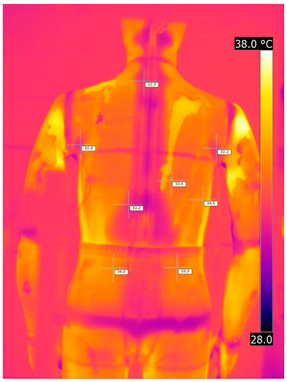	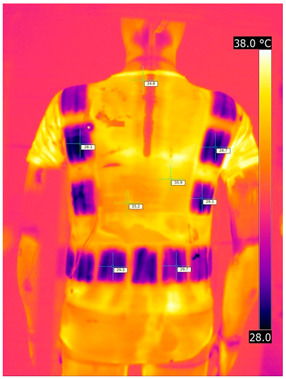	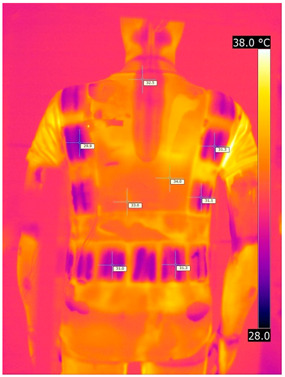

**Table 3:** Pictures from thermal imaging cameras for variants III and IV.

**Variant III_ Undergarment A without PCM with Outerwear**		
Before putting on T-shirt	Directly after putting on T-shirt	10 min after the putting on T-shirt
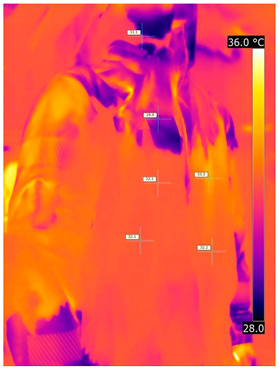	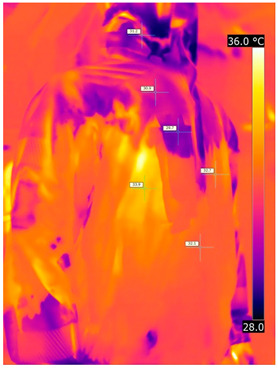	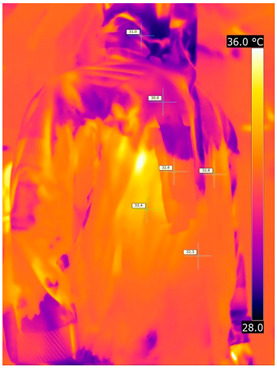
**Variant IV_ Undergarment A with PCM with Outerwear**		
Before putting on T-shirt	Directly after putting on T-shirt	10 min after the putting on T-shirt
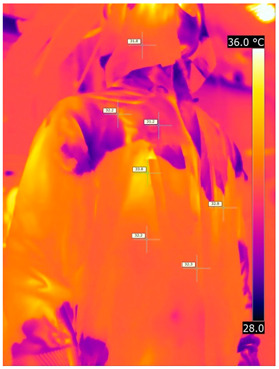	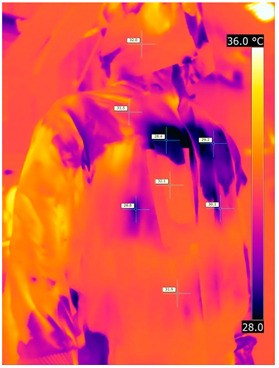	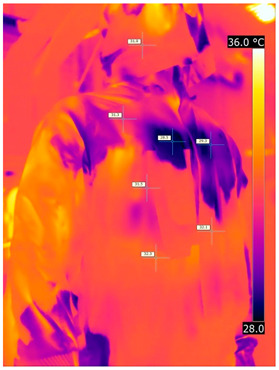
**Variant III_ Undergarment A without PCM with Outerwear**		
Before putting on T-shirt	Directly after putting on T-shirt	10 min after the putting on T-shirt
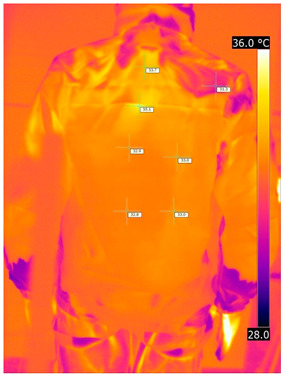	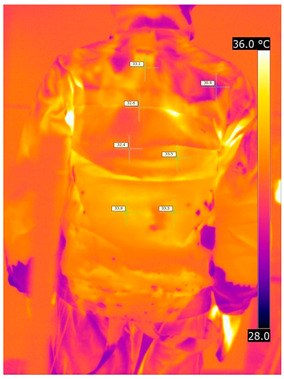	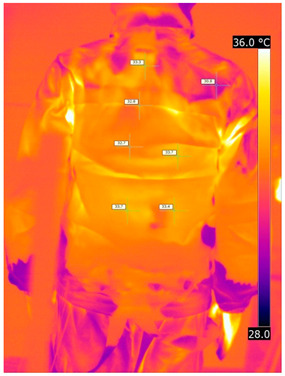
**Variant IV_ Undergarment A with PCM with Outerwear**		
Before putting on T-shirt	Directly after putting on T-shirt	10 min after the putting on T-shirt
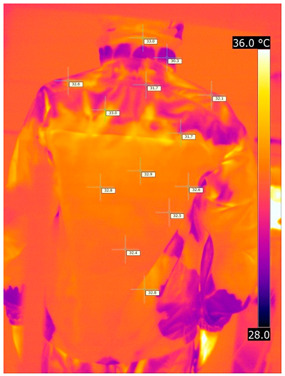	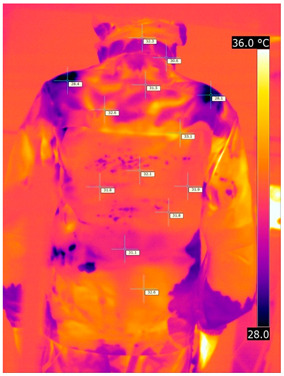	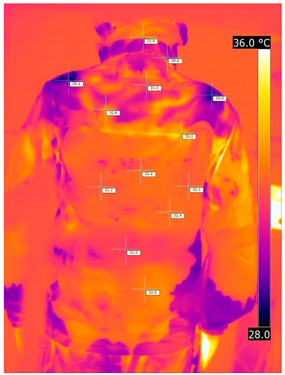

## Data Availability

Not applicable.
